# A Low-Delay Dynamic Range Compression and Contrast Enhancement Algorithm Based on an Uncooled Infrared Sensor with Local Optimal Contrast

**DOI:** 10.3390/s23218860

**Published:** 2023-10-31

**Authors:** Youpan Zhu, Yongkang Zhou, Weiqi Jin, Li Zhang, Guanlin Wu, Yiping Shao

**Affiliations:** 1 School of Optics and Photonics, Beijing Institute of Technology, 5 South Zhongguancun Street, Haidian District, Beijing 100081, China; zhuypbit@aliyun.com (Y.Z.);; 2 Kunming Institute of Physics, No. 31, Jiaochang East Road, Wuhua District, Kunming 650221, China; zyk1120102464@163.com (Y.Z.);; 3 School of Life Science, Beijing Institute of Technology, 5 South Zhongguancun Street, Haidian District, Beijing 100081, China; 4 College of Mechanical Engineering, Zhejiang University of Technology, Hangzhou 310023, China

**Keywords:** infrared image, dynamic range compression, local optimal contrast, real-time imaging

## Abstract

Real-time compression of images with a high dynamic range into those with a low dynamic range while preserving the maximum amount of detail is still a critical technology in infrared image processing. We propose a dynamic range compression and enhancement algorithm for infrared images with local optimal contrast (DRCE-LOC). The algorithm has four steps. The first involves blocking the original image to determine the optimal stretching coefficient by using the information of the local block. In the second, the algorithm combines the original image with a low-pass filter to create the background and detailed layers, compressing the background layer with a dynamic range of adaptive gain, and enhancing the detailed layer for the visual characteristics of the human eye. Third, the original image was used as input, the compressed background layer was used as a brightness-guided image, and the local optimal stretching coefficient was used for dynamic range compression. Fourth, an 8-bit image was created (from typical 14-bit input) by merging the enhanced details and the compressed background. Implemented on FPGA, it used 2.2554 Mb of Block RAM, five dividers, and a root calculator with a total image delay of 0.018 s. The study analyzed mainstream algorithms in various scenarios (rich scenes, small targets, and indoor scenes), confirming the proposed algorithm’s superiority in real-time processing, resource utilization, preservation of the image’s details, and visual effects.

## 1. Introduction

Because of its advantages for passive imaging and night imaging, infrared imaging systems have been widely used in military, aerospace, security, and other fields. It has always been a goal to obtain high-quality imaging results. Infrared imaging systems typically use 14-bit or higher ADC (analog to digital converter) acquisition circuits to gather information from an infrared scene with a high dynamic range, while the majority of image display devices are 8-bit displays with a low dynamic range. This may result in some details being lost during the display process. To combine the application scenarios of infrared imaging devices in order to better adapt them to the visual characteristics of the human eye, we need to perform high-dynamic-range compression of the image. Of course, the loss of information from the image in this process is inevitable, but different processing methods will exhibit different details, and the effect of the processing method depends mostly on human vision. In addition, in recent years, uncooled infrared sensor technology has been significantly improved, and the applications have become increasingly widespread, especially in civilian products. Therefore, how to compress images with a high dynamic range into those with a low dynamic range while preserving the majority of the detailed information is one of the critical technologies for infrared systems; at the same time, low-delay image processing is also essential for back-end target detection and tracking.

Contrast refers to the measurement of different brightness levels between the brightest white and the darkest black in the light and dark areas of an image. The greater the difference, the greater the contrast, and the smaller the difference, the lower the contrast. It can be global or local (i.e., concentrated in a small area).

Appropriate local contrast can make the image present a concave and convex three-dimensional sense, which can improve the visual effect of the human eye. In addition, the enhancement of local contrast can minimize the halo effect, making the picture clearer.

To address the problem of dynamic range compression, many researchers have developed algorithms for displaying high dynamic ranges and enhancing details [1]. Overall, high-dynamic-range image compression algorithms can be classified into traditional mapping-based algorithms, gradient domain-based compression algorithms, and image layering-based compression algorithms. Besides these, there are also a few methods [2,3] that cannot be classified into any of these three classes. Different algorithms are applicable for various application contexts and vary in their complexity and performance.

### Related Work

Mapping-based high-dynamic-range IR (infrared radiation) image enhancement algorithms are the simplest and most widely used, and the visual effect of the human eye is obviously improved. These algorithms include self-gain-based linear mapping, Gamma curve correction, histogram projection, etc. In order to make the distribution of the histogram as uniform as possible, the earliest method of histogram equalization redistributed the image’s grayscale using a cumulative distribution function. A uniform probability density distribution can be obtained using this method, but there are drawbacks as well, including overenhancement, higher noise levels, the loss of some details, and fading. To solve these issues, a threshold-based plateau histogram equalization (PHE) algorithm was proposed in the literature [4]. The plateau histogram modifies the ordinary histogram algorithms by adding a threshold. When the density of each gray value exceeds the threshold, it will be processed, thereby improving the global contrast and avoiding a small number of outliers affecting the global distribution of the gradient. The literature [5] has proposed an adaptive histogram equalization algorithm (AHE), which computes a histogram function based on a local window and can enhance the local contrast of an image while further highlighting its finer details. However, the AHE algorithm is prone to creating a great deal of noise. In light of this, a contrast-limited adaptive histogram equalization algorithm (CLAHE) was proposed in the literature [6], which reduces the amplitude of the noise with a clipping threshold. Some researchers [7,8] have also made some improvements to the CLAHE algorithm. Generally, the self-gain-based equalization algorithms perform noticeably worse than the histogram-based equalization algorithms. Nevertheless, the histogram-based algorithms lack flexibility in some particular applications and are also susceptible to overenhancement, block effects, etc., because they are entirely dependent on the histogram’s statistics.

A gradient dynamic range compression algorithm compresses the large-scale gradient and keeps the low-amplitude information. Fattal [9] proposed an algorithm as the framework of the gradient-domain-based high-dynamic-range image compression algorithm (GDHDRC). Based on this, a detail-preserving algorithm (GDHDRC-DPS) was proposed, and further research [10,11,12,13] refined the GDHDRC-DPS algorithms even more. On most occasions, these algorithms produce rather acceptable outcomes; however, there are significant limitations in certain occasions. On the one hand, the factors used to smooth the data items and gradient items need to be properly designed in order to prevent oversaturation; on the other hand, the details of the output images are still not sharp enough to meet some of the requirements of displaying infrared images with a HDR (high dynamic range).

**Figure 1:**
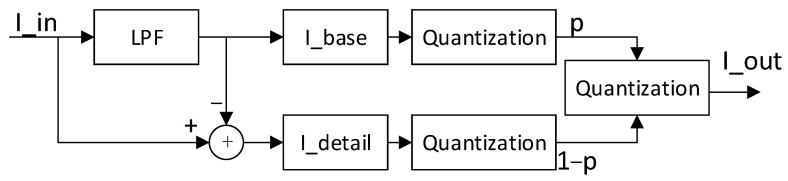
Framework of the dynamic range compression algorithm based on image layering. LPF, low-pass filter; I_base, the background layer image; I_detail, the detailed layer image; p, parameter.

The algorithms based on image layering [14,15,16,17,18,19,20,21,22,23,24,25,26,27] decompose the original image into various components, such as the background layer of the image and the detailed layer of the image, and then process each component separately; afterwards, the compressed background layer image and the stretched detailed layer image are reintegrated into an image in which the dynamic range is compressed and the detailed layer image is enhanced. The basic framework is shown in Figure 1, where the original image is decomposed into a background layer image and a detailed layer image after being processed by a low-pass filter; the background layer image undergoes dynamic range compression, and the detailed layer image is enhanced. A final image with a low dynamic range is obtained after re-fusing. Although the choice of algorithms can differ for low-pass filtering, the background layer, the detailed layer, and image fusion, they are basically improved and promoted in this framework.

Specifically, an infrared image enhancement algorithm based on bilateral filtering (bilateral filter and dynamic range partitioning; BF-DRP) was proposed in the literature [14]. The algorithm divides the original image into a background layer image and a detailed layer image using bilateral filtering, then performs Gamma curve processing (compression or expansion, respectively) on the background layer image and the detailed layer image, and finally reintegrates the background layer and the detailed layer image to obtain the detail-enhanced image. The bilateral filter is a nonlinear filter that takes both the spatial proximity and the pixels’ grayscale difference into account, smoothing out uniform areas of the input image such as the sky and sea while preserving strong edges such as floors. However, the drawbacks of the BF-DRP algorithm are also obvious: (1) several parameters of the algorithm require fine-tuning to achieve better enhancement; (2) the original image minus the image after bilateral filtering is used to obtain a detailed layer image, the edges of which will be sharper than the edges of the original image or even have gradient inversion, while the highlighted noise appears in flat areas [15,16].

Based on this, an improved version of the BF-DRP algorithm, namely the bilateral filter and digital detail enhancement (BF-DDE) algorithm, was proposed in the literature [17,18], with the goal of obtaining a corrected background layer image that was closer to the original image. In addition, research on the human visual system [17] showed that human eyes are more sensitive to noise in uniform regions than in complex regions. The literature [19] first described the noise mask function, and the literature [20] used a noise mask function based on local variance, both of which clearly improved the image’s overall contrast and improved the information on the target and the detail. The algorithms based on a bilateral filter have two problems: (1) the detailed layer image obtained with the bilateral filter is prone to gradient flipping because the mean filter method based on Gaussian weights is unstable when one pixel has a large difference from its adjacent pixels; (2) the computational complexity of bilateral filter is O(Nr2), and as the filter window increases, the computational time will increase quadratically. To solve these problems, a linear filter, i.e., a guided filter (GF), for the detail-enhancement algorithm was proposed in the literature [21], while the studies of both [22,23] introduced an algorithm for enhancing images with a high dynamic range (GF-DDE) based on a guided filter. The guided filter was used instead of the bilateral filter in the GF-DDE method, which significantly decreased the algorithm’s computing complexity but worsened its edge retention. An algorithm for enhancing images with a high dynamic range (LEPF-DDE) based on a local edge-preserving filter was proposed by [24]. The local edge-preserving filter (LEPF) was used to separate the original image into a background layer image and one or more detailed layer images. Next, the multi-scale background layer image undergoes maximum entropy-based Gamma curve correction, and the detailed layer undergoes of elimination of the artifacts and amplification of the detail. Finally, the detailed layer image and the background layer image are re-bounded. An edge-preserving filter algorithm called LEPF has been proposed in the literature [24,25], which aimed to filter low-amplitude noise while maintaining strong edges.

On the whole, the traditional image mapping method has a general effect on the retention of detail and image contrast, in which the gradient compression algorithm retains the details better and the contrast is enhanced, but the parameters need to be carefully adjusted for different scenes. The algorithm based on image layering results in greater improvement in the retention of detail and noise suppression, but traditional methods are still used in the background layer, and the contrast enhancement effect is general and can easily cause halos and other problems.

The DRCE-LOC algorithm proposed in this study focused on improving image delay, storage resources, FPGA-based implementation, and the trade-off between global contrast and local contrast while applying the framework of dynamic range compression based on image layering (Figure 1).

At the same time, the algorithm was optimized and implemented on an FPGA (field-programmable gate array). For the low-delay image processing requirements of offline applications [26], it is often necessary to design a hardware processing method based on an FPGA. This is different from many CPU (central processing unit)-based algorithms. Multiplication can be implemented on a parallel pipelined FPGA with only one clock cycle, but exponential calculation that is simple on a CPU is difficult to implement on an FPGA. Therefore, it is necessary to effectively utilize the characteristics of the FPGA to study the design and improvement of algorithms. We conducted experiments on an infrared imager equipped with the FPGA and uncooled infrared sensors and evaluated the effect of the algorithm.

## 2. Methods

### 2.1. DRCE-LOC

An uncooled infrared sensor does not need a refrigeration device and can work at room temperature. It has many advantages, such as fast start-up, low power consumption, small size, light weight, long life, and low cost. In recent years, such sensors have been widely used in military and civil night vision products. The proposed algorithm was applied to an infrared imager equipped with 1024 × 768 uncooled infrared sensors, and all experiments were carried out on this system.

**Figure 2:**
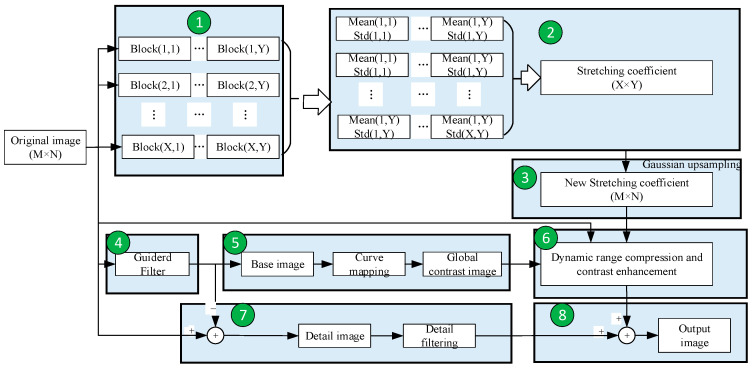
Framework of the DRCE-LOC algorithm.

The framework of the DRCE-LOC algorithm is shown in Figure 2. Its basic principle is as follows. Firstly, the brightness value and contrast value are re-corrected in each local block to achieve the best possible local contrast. Secondly, the Gaussian model [28] is used to smooth the contrast value of the local block to eliminate the block effect. Then, a brightness adjustment algorithm guided by the global brightness is applied. The algorithm uses the background layer obtained by the guided filter to obtain the brightness-guided image after passing through the global image. This step maintains the brightness relationship of the original image as much as possible. Finally, the detailed layer obtained via the guided filter and the image obtained after brightness and contrast correction are re-fused to obtain the final image. The process of implementing the algorithm includes the following eight steps.

- Step 1: Image segmentation.

To reduce the amount of storage, the original image $I_{in}$ (with a size of M×N) is divided into X×Y local blocks. The size of each local block is $\frac{M}{X}\times \frac{N}{Y}$. The mean value (${Block}_{mean}$) and its standard deviation are calculated. ${Block}_{mean}$ reflects the level of brightness of local blocks in the original image, and ${Block}_{std}$ reflects the level of richness of the details of local blocks in the original image. The sizes of ${Block}_{mean}$ and ${Block}_{std}$ are both X×Y.

- Step 2: Calculation of the local block’s stretching coefficient.

The stretching coefficient of each local block is determined by the standard deviation of each local block, as shown in Equation (1)

(1) $$Stretch_{para}=\frac{255}{Block_{std}+Bias}$$

where ${Block}_{str}$ is the stretching coefficient of the local block and *Bias* is a constant set to limit the coefficient of gain from being too large. After processing, the size of ${Block}_{str}$ is X×Y.

- Step 3: Upsampling of the parameters of the local block.

**Figure 3:**
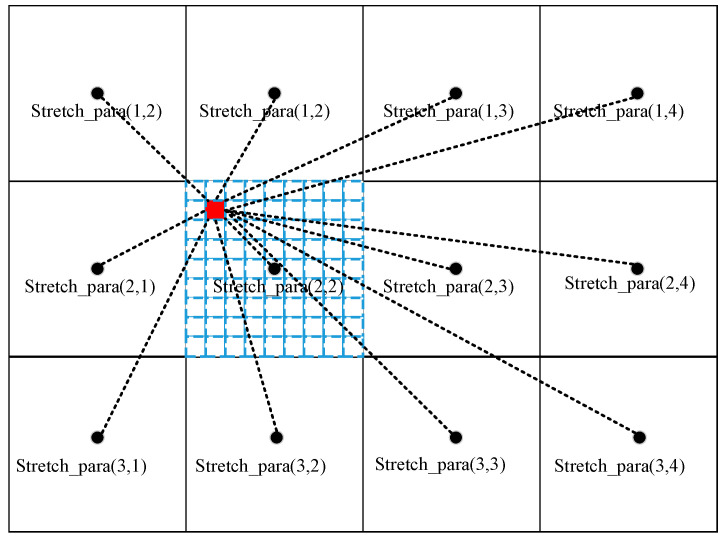
Schematic of the image upsampling process.

${Stretch}_{para}$ and ${Block}_{str}$ are upsampled into M×N dimensions, the basic principle of which is shown in Figure 3. The stretching parameter and the mean value of each local block are projected to the center of the local block of the original image, and the stretching parameter and mean parameter are calculated at the position of each pixel in the original image. They are calculated from the parameters of each local block and the Gaussian weights of the distance to the center of each local block.

(2) $$NStretch_{para}(i,j)=\frac{\sum_{x=1}^{X}\sum_{y=1}^{Y}Stretch_{para}(x,y)*\exp(-\frac{d_{\left(i,j)(x,y\right)}{}^{2}}{\delta^{2}})}{\sum_{x=1}^{X}\sum_{y=1}^{Y}\exp(-\frac{d_{\left(i,j)(x,y\right)}{}^{2}}{\delta^{2}})}$$

(3) $$NMean_{para}(i,j)=\frac{\sum_{x=1}^{X}\sum_{y=1}^{Y}Block_{mean}(x,y)*\exp(-\frac{d_{\left(i,j)(x,y\right)}{}^{2}}{\delta^{2}})}{\sum_{x=1}^{X}\sum_{y=1}^{Y}\exp(-\frac{d_{\left(i,j)(x,y\right)}{}^{2}}{\delta^{2}})}$$

where ${NStretch}_{para}$ and ${NMean}_{para}$ are the stretching parameter and the mean parameter after upsampling, respectively, and their sizes are both M×N; $\delta^{2}$ is a constant used to control the Gaussian weights; and $d_{\left(i,j)(x,y\right)}$ represents the distance from the coordinates of the original image (i,j) to the coordinates of the local block’s center (x,y).

- Step 4: Layering of the guided filter.

Guided filters [19] are edge-preserving linear filters that have been widely used in image enhancement. Here, they are used to divide the original image into a background layer image and a detailed layer image. We assumed that the input image is p, the output image is q, and the guide image is I. The fundamental idea is to combine an original image with a guide image to produce a filtered image that resembles the guide image. The equation is displayed in Equation (4)

(4) $$\left\{ \begin{array}{l} q=a_{k}I+b_{k},\forall i\in w_{k}\\ a_{k}=\frac{\bar{p_{k}I_{k}}-\bar{p_{k}}\bar{I_{k}}}{\bar{I_{k}{}^{2}}-{\bar{I_{k}}}^{2}+\varepsilon }=\frac{\frac{1}{\left|w\right|}\sum_{i\in w_{k}}I_{i}p_{i}-u_{k}\bar{p_{k}}}{\sigma_{k}{}^{2}+\varepsilon }\\ b_{k}=\bar{p_{k}}-a_{k}u_{k} \end{array}\right.$$

where $w_{k}$ is a square window with a diameter of w with k as the center; |*w*| represents the number of the pixels in the window; $a_{k}\;$and $b_{k}$ are linear coefficients and are constants in the local window $w_{k}$; and i denotes the number of pixels in $w_{k}$. It should be ensured that the linear coefficient satisfies the formula above so that the difference between q and p is minimized.

- Step 5: Calculation of the brightness guide map.

In order to increase the computational speed of the FPGA and maintain the light–dark relationship of the original image, a linear mapping method is used. Gamma curves, histogram equalization, and other methods can be used in some specialized applications. The mapping method is shown in Equation (5)

(5) $$I_{gc}(i,j)=\frac{255*(I_{in}(i,j)-\bar{I_{in}})}{Std(I_{in})+\lambda }+Bright$$

where $Std{(I}_{in})$ is the standard deviation of the original image, *Bright* is the desired brightness of the image, *λ* is a constant to avoid excessive gain, and $I_{gc\;}(i,j)$ is the mapped 8-bit global brightness guide.

- Step 6: Dynamic range compression and contrast enhancement.

Four factors are needed for dynamic range compression and contrast enhancement: $I_{in}$, $I_{gc\;}$, ${NMean}_{para}$, and ${NStretch}_{para}$. The corresponding equation is

(6) $$Base_{out}(i,j)=k1\times NStretch_{para}\times \left[I_{in}(i,j)-NMean_{para}(i,j)\right]+k2\times I_{gc}$$

where ${Base}_{out}(i,j)$ is the output image after contrast enhancement, k1 is the parameter that controls the local contrast, k2 is the parameter that controls the global contrast, $I_{gc\;}$ is the brightness-guided image calculated in Step 5, and ${NMean}_{para}$ and ${NStretch}_{para}$ are the local mean parameters and stretching parameters calculated in Step 3, respectively.

- Step 7: Filtering of the detailed layer.

The detailed layer obtained through the previous steps contains noise. A noise mask function based on human vision [17] is utilized to reduce noise. The basic principle of the function is that the human eye is sensitive to noise in a uniform scene but is insensitive to noise in areas with large amounts of detail.

(7) $$Detail_{out}=Detail_{image}\times \left|a_{k}\right|*\left[g_{L}+(g_{H}-g_{L})\right]$$

where $Detail_{image}$ is the detailed layer image obtained after layering of the guided filter and $a_{k}$ is the parameter that was calculated during the process of layering the guided filter, which reflects the quantity of local information.

- Step 8: Image enhancement.

The final image is obtained by re-integrating the contrast-enhanced image and the detailed layer image.

(8) $$I_{out}=Base_{out}+DDE\times Detail_{out}$$

where $I_{out}$ is the final output image and DDE is the detail enhancement factor.

### 2.2. Noise Suppression

**Figure 4:**
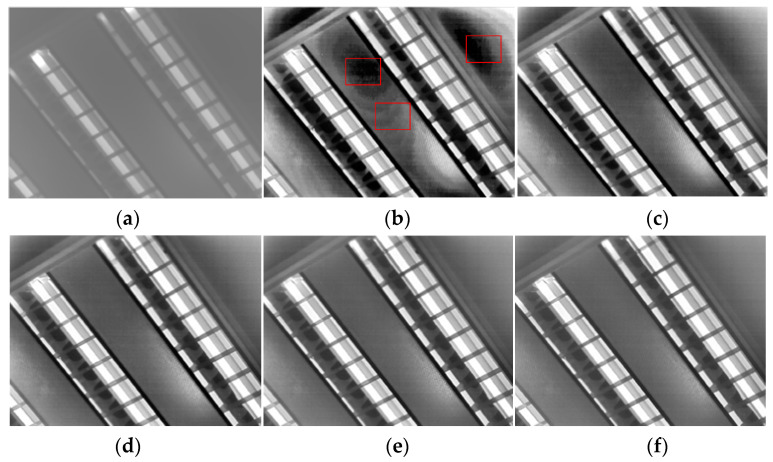
Effects of local noise suppression. (**a**) AGC; (**b**) $I_{gc}$ = 128, bias = 0; (**c**) $I_{gc}$ = 128, bias = 128; (**d**) $I_{gc}$ = 128, bias = 256; (**e**) $I_{gc}$ = 128, bias = 384; (**f**) $I_{gc}$ = 128, bias = 512.

The algorithm includes two sources of noise: noise in the detailed layer and noise in the background layer. The detailed layer’s noise adopts the principle of [17]. The over-enhancement of the local contrast, which is primarily the source of the noise in the background layer, is suppressed by *Bias* in Equation (1). In order to analyze the influence of stretching the background layer on local noise, we set $I_{gc}=128$. At this point, the stretching of the image in terms of global contrast is 0, and all the noise in the image comes from the stretching of local contrast. The algorithm next divides the 1024 × 768 infrared images into 4 × 3 squares and set $\delta^{2}$ to 3600 (the selection of this parameter is introduced in the next section). Then, the effect of *Base_out* imaging under different values of bias is shown in Figure 4.

AGC (adaptive gain control) is a method of compressing high dynamic ranges that maps the dynamic range of an input image to a specified dynamic range. Since a 14-bit image cannot be visualized on most display devices, we considered the image processed by AGC as the input image and compared it with images processed by other algorithms.

Figure 4 illustrates how the final image’s noise level in terms of contrast varied significantly with various biases. The image in the uniform area of the scene was overamplified when Bias=0, producing a significant amount of noise. The noise in the uniform region steadily reduced with an increase in the bias, while the local contrast of the image diminished with the noise. The image’s noise was smaller and the local contrast performed better when the bias was around 256.

### 2.3. Halo Suppression and Contrast Control

**Figure 5:**
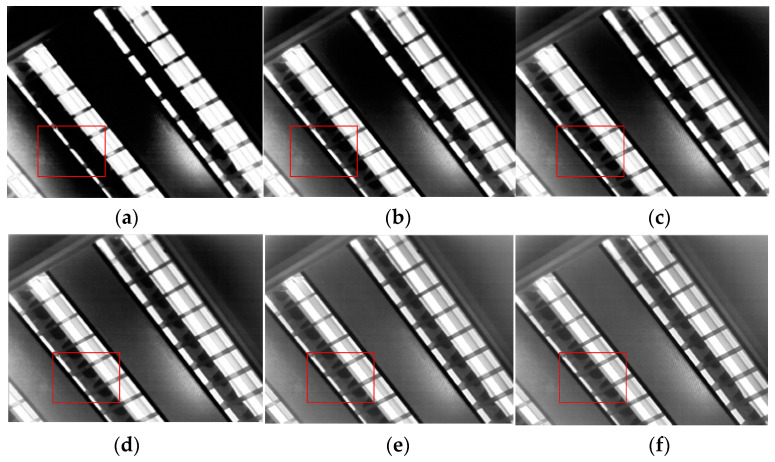
The halo suppression effect under different values of k2. (**a**) *k*2 = 1.5; (**b**) *k*2 = 1; (**c**) *k*2 = 0.8; (**d**) *k*2 = 0.5; (**e**) *k*2 = 0.2; (**f**) *k*2 = 0.1.

A halo is a virtual shadow that extends from the edge of the image, which is a problem that can easily happen during image blocking. Suppressing halos can keep the image in its original state. In this study, we combined local and global information, but the halo problem could also exist in the case of improper parameter selection. In Equation (6), k2 was used to control the halo problem and the global contrast. When k2=0, the algorithm became a stretching algorithm with pure local contrast; as k2 increased, the weight of global contrast in the final image gradually increased. In particular, in Equation (6), the mean component of the local stretched image was eliminated rather than the algorithm just adding the local stretched image and the global image. Therefore, the global image plays a role in guiding the global contrast in Equation (6). The final image’s global contrast will be closer to the global compressed image as k2 increases, and the halo will be weaker; however, better local contrast can be achieved by controlling k1. Figure 5 shows the effect of the algorithm under different values of k2 (k1=1; the basis for selecting this parameter is presented in the next section). The red rectangles in the images facilitated a comparison of the halo suppression effect under different values of k. It can be seen from Figure 5 that when k2 is large, the contrast of the final image was excessively high and some local information was even obscured, as shown in Figure 5a. As k2 increased, the overall contrast of the image steadily declined, and when k2=0.1, the brightness of the local blocks of the image was nearly the same, losing the overall brightness and the blackness of the original image. When k2=0.1, if a continuous video sequence enters the algorithm module proposed in this study, a transformed halo will develop as a result of the alteration of the local information. It can be seen from Figure 5d that when k2=0.5 or so, the final image is nearly identical to the global contrast image, and the halo phenomenon almost disappears, whereas improved global contrast and local contrast were achieved.

### 2.4. Parameter Settings

The choice of parameters determines how well the algorithm performs. The selection of the parameters in the algorithm was as follows:

$\frac{M}{X}\times \frac{N}{Y}$ is the local block size of the image. In general, the smaller the local block size is, the better the local contrast will be. The computational effort will rise as the local block size falls, since more local blocks are required. Tiny squares also have a tendency to bring about localized halo alterations because of the movement of items in real-world settings. For continuous video, a value of $\frac{M}{X}\times \frac{N}{Y}=64\times 64$ achieves better results.

Bias is used to suppress the local overenhancement phenomenon, which is described in Section 2.2, and generally achieves better results when it is between 128 and 384.

The parameter $\delta^{2}$ is used to control the Gaussian kernels’ curvature during downsampling and upsampling of the image. The selection of $\delta^{2}$ is strongly influenced by the size of the image block. Better results are achieved when $\delta^{2}\geq \frac{M}{X}\times \frac{N}{Y}$, which eliminates the obvious block effect that may be present in the image.

The parameter λ is set to prevent the global contrast from being overenhanced. It performs the same function as bias, with the exception that bias controls the local contrast while the global contrast is controlled when the parameter is modified. Similarly, better results are achieved when λ is between 128 and 384.

The parameter k1 is used to control the local contrast. Since Equation (2) already places a limit on the range of the image, higher results can be achieved when k1=1.

The parameter k2 is used to suppress halos and control the global contrast. A detailed description is given in Section 2.3. Better results can be achieved when k2 is in the range of 0.2 and 0.7.

### 2.5. Complexity of the Algorithm

The algorithm proposed in this study is built on a framework based on guided filter layering, which does not make the original guided filter any more computationally complex. Similarly, the computational complexity of the algorithm proposed in this study is O(n) and depends solely on the number of pixels. However, the following improvements can be applied during the construction of the algorithm, particularly for real-time videos, to further reduce the method’s resource consumption:

- Calculating the local information of the image local blocks, which can be achieved by using the local information of the data of the previous image of consecutive frames, thus achieving an algorithm with a delay of less than one frame while storing the complete image frame differently.
- In the calculations of upsampling and downsampling of the image, the Gaussian kernel can be saved in advance as a parameter to avoid exponential calculations during the execution of the algorithm.

## 3. Results and Discussion

### 3.1. Quantitative Assessment

To analyze and evaluate the effect of the algorithm, four image indicators, namely the root mean square difference [29] (root mean square, RMS), image entropy [30], structural similarity [31] (SSIM), and the Tenengrad clarity index [32], and the algorithm’s running time were used. For calculating the running time, the experiment was carried out in the same environment as follows: operating system, Windows 11; CPU, 12th Gen Intel (R) Core (TM) i9-12900HX 2.30 GHz; RAM, 32.0 GB.

RMS is defined as

(9) $$RMS=\sqrt{\frac{1}{MN}\sum_{i=1}^{M}\sum_{j=1}^{N}{\left(I(i,j)-\bar{I}\right)}^{2}}$$

where I denotes the image to be evaluated, $\bar{I}\;$represents the mean value of the image, M is the width of the image, and *N* is the height of the image. The larger the RMS, the better the overall contrast of the image.

Image entropy is defined as

(10) $$Entropy=-\sum_{i=0}^{255}p_{i}\log p_{i}$$

where $p_{i\;}$represents the probability of each grayscale value. Greater entropy indicates that the grayscale has been stretched further.

SSIM is defined as

(11) $$SSIM(I,ref)=\frac{\left(2u_{I}u_{ref}+C_{1})(2\sigma_{ref}+C_{2}\right)}{\left(u_{I}{}^{2}+u_{ref}{}^{2}+C_{1})(\sigma_{I}{}^{2}+\sigma_{ref}{}^{2}+C_{2}\right)}$$

where ref represents the reference image, I is the image obtained by stretching the original image with adaptive gain, u represents the mean value, σ represents the standard deviation, and $C_{1}\;$and $C_{2}\;$are very small numbers to prevent the denominator from becoming 0. A larger SSIM indicates that the two images are more similar in structure.

Tenengrad is a gradient-based function that extracts the gradient values in horizontal and vertical directions through the Sobel operator [33]. It is defined as

(12) $$S(i,j)=\sqrt{G_{x}*I(i,j)+G_{y}*I(i,j)}$$

(13) $$Tenengrad=\frac{1}{MN}\sum_{i=1}^{M}\sum_{j=1}^{N}S{\left(i,j\right)}^{2}$$

(14) $$G_{x}=\frac{1}{4}\left[\begin{array}{ccc} -1 & 0 & 1\\ -2 & 0 & 2\\ -1 & 0 & 1 \end{array}\right],G_{y}=\frac{1}{4}\left[\begin{array}{ccc} 1 & 2 & 1\\ 0 & 0 & 0\\ -1 & -2 & -1 \end{array}\right]$$

where S(i,j) represents the gradient of Image I at the point (i,j); $G_{x}$ and $G_{y}\;$represent the Sobel convolution kernels in the horizontal direction and vertical direction, respectively. A larger Tenengrad means a clearer image.

**Figure 6:**
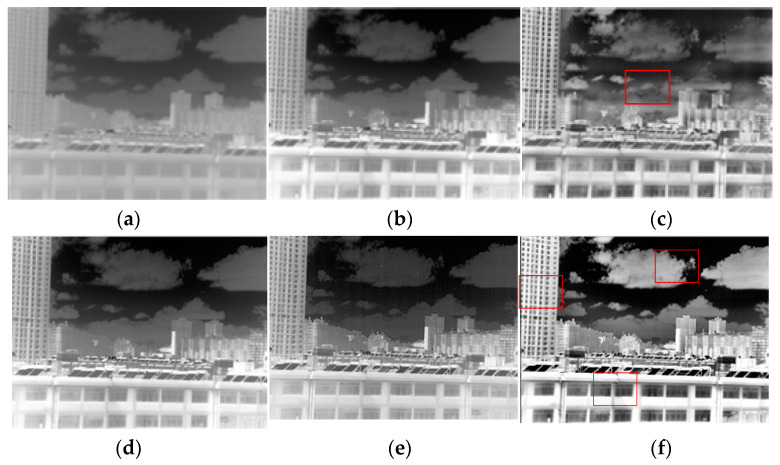
Comparison of the effects for the rich scene (Scene 1). (**a**) AGC; (**b**) HE; (**c**) CLAHE; (**d**) GF-DDE; (**e**) BF-DRP; (**f**) proposed method.

**Figure 7:**
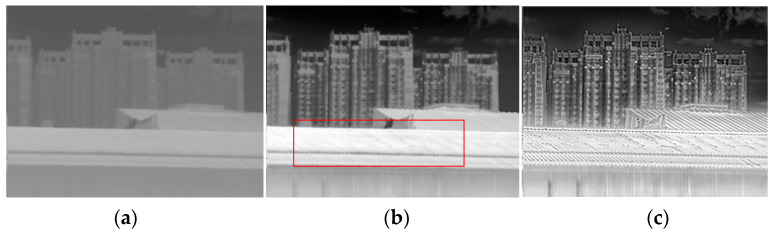

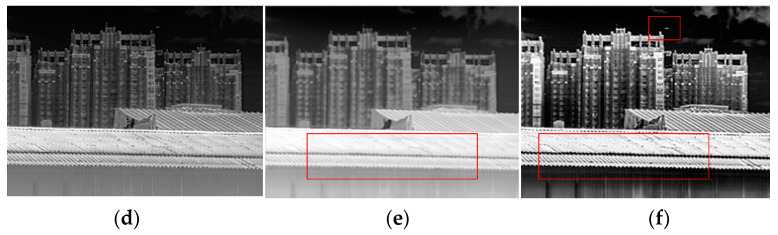
Comparison of the effects for the scene with a small target scene (Scene 2). (**a**) AGC; (**b**) HE; (**c**) CLAHE; (**d**) GF-DDE; (**e**) BF-DRP; (**f**) proposed method.

**Figure 8:**
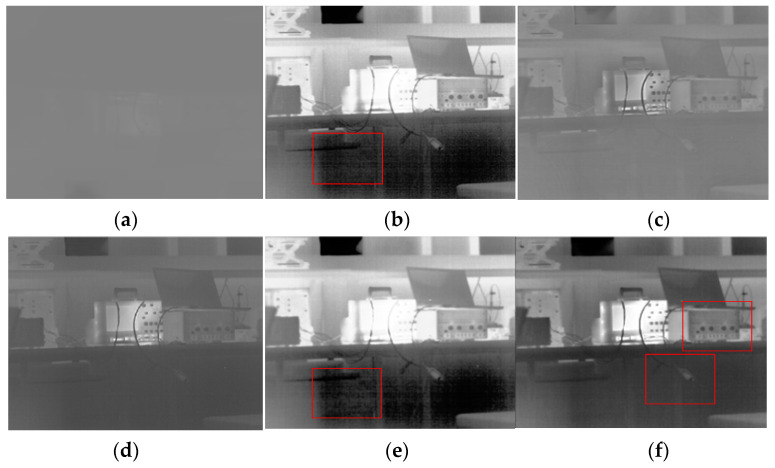
Comparison of the effects for the indoor scene (Scene 3). (**a**) AGC; (**b**) HE; (**c**) CLAHE; (**d**) GF-DDE; (**e**) BF-DRP; (**f**) proposed method.

To evaluate the processing effect of the DRCE-LOC algorithm in this study, we used the typical AGC, HE, CLAHE, GF-DDE, and BF-DRP algorithms as a comparison in three typical scenarios: a rich scene, a scene with a small target, and an indoor scene. The following can be seen from Figure 6, Figure 7 and Figure 8.

(1) The CLAHE algorithm and the proposed algorithm had the best results in terms of image local contrast. CLAHE had two problems. First, it easily produced an overenhancement phenomenon, leading to the noise being amplified, as seen in Figure 6c, where the red rectangle has a significant amount of noise. Second, the scene’s energy was weak when the overall contrast was low, as shown in Figure 7c, where the thermal radiation of the indoor scene was weak and the overall contrast of the whole image was low.

(2) The proposed algorithm had the best results in terms of retaining small targets and details, as shown in Figure 6, where the two small dots at the top right are aircraft targets. The proposed algorithm could highlight the aircraft targets and other detailed information without overexposure.

(3) The proposed algorithm had the best global contrast, as shown in Figure 6 and Figure 8. As shown in Figure 6, each algorithm maintained the details well, but the proposed algorithm was more transparent and more informative. In Figure 8, HE, BF-HDR, and the proposed algorithm all had good global contrast, but HE and BF-HDR both showed overenhancement. The proposed algorithm achieved better global contrast while suppressing overenhancement.

Table 1 shows the results of the evaluation of the three scenarios shown in Figure 6, Figure 7 and Figure 8. It can be seen from the table that for the RMS index, no algorithm showed particularly good superiority; that is, several algorithms had no significant difference in the global contrast. For entropy, the HE algorithm performed best in all three scenarios; that is, the distribution of gray in the image after HE processing was the most uniform, which was determined by the principle of the HE algorithm. However, the HE-based algorithm is prone to excessive stretching, which can also be seen in Figure 6, Figure 7 and Figure 8. For the SSIM index, the closer it is to 1, the greater the similarity to the original image. It can be seen from the index that the performance of several algorithms was not very good. This is because the original image was a 14-bit image, and the image’s structure underwent changes after compression. Tenengrad represents the clarity of the image. The proposed algorithm showed very obvious superiority in all three scenarios, indicating that the proposed algorithm retained the most detailed information and had the best image clarity after dynamic range compression.

In terms of the operation time, the algorithm based on AGC had the least delay, followed by the algorithm proposed in this study. The AGC algorithm had the worst enhancement effects for all image indicators. Therefore, the algorithm proposed in this study has obvious advantages in terms of the operation time.

### 3.2. Implementation of the Algorithm on an FPGA

**Figure 9:**
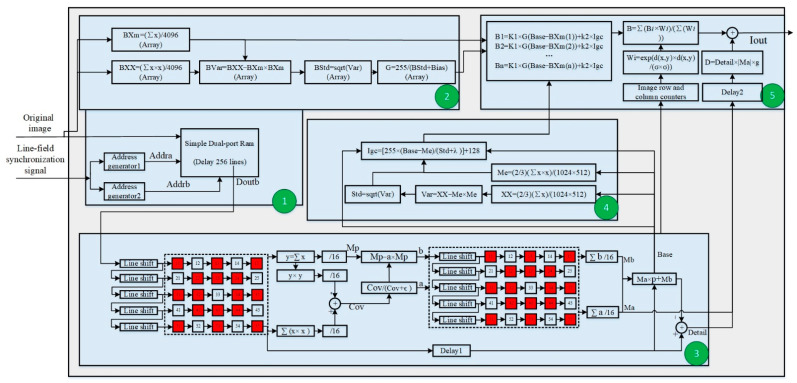
Diagram of FPGA-based implementation of the algorithm.

The proposed algorithm’s FPGA implementation is depicted in Figure 9. This is made up of five parts, each of which is indicated by a green circle. The algorithm is applied to a 1024 × 768 infrared imager with an image frame rate of 25 Hz, a pixel clock of 30 MHz, and 200 pixel clocks of line fading. The algorithm was implemented on the Xilinx (San Jose, CA, USA) A7 FPGA chip and was written in VHDL. Each component’s implementation and resource utilization were examined in turn.

Part 1: The first part was to finish the process of image buffering in 256 lines so that the calculation of local image stretching and the bias factor needed to wait for 256 lines (in a local block of 64, where $\delta^{2}=25$). This module’s operation was straightforward and only required 1 Mb of Block RAM, but it consumed the most resources and had the longest image delay, with a total of 2.1875 Mb of Block RAM and an image delay of 313,344 pixel clocks.

Part 2: The second part was to calculate the pull-up and bias coefficients of the image’s local blocks. Each local block had 4096 pixels and was 64 × 64 pixels in size. There were 192 local blocks in a single image frame. To store the values of data accumulation, square accumulation, and the gain coefficient within the local block, three arrays of 192 bits each were built by the computation module. The data were 26 bits wide. The image was shifted 12 bits to the right after all the data in the local block had been calculated to produce the image’s mean value for the local block (BXm) and the mean image variance (BXX). The module accumulated the image data according to the row and column numbers corresponding to the relevant positions in the arrays; furthermore, the local block variance Bstd was computed by calculating the difference between the square of BXm and BXX, and the local standard deviation was generated by taking the square root of Bstd. Finally, the gain coefficient of the local block G could be calculated by Equation (1). A divider was needed for this stage. Overall, this part used resources of 0.0142 Mb of Block RAM, a divider, and some other auxiliary computing resources. The local block calculation required a delay of 64 rows of data, and the calculation of the mean, variance, standard deviation, gain coefficient, and algorithm required 1, 1, 34, and 26 pixel clocks, respectively, with a total delay of 78,398 pixel clocks or about 0.0026 s. Moreover, since Part 1 and Part 2 can run simultaneously, the system was not subjected to any additional delay.

Part 3: The third part was image layering based on a guided filter, which used a 5 × 5 window, and the data stream was be divided into two main steps:

(1) Calculate the coefficients a,b. To create a real-time image window of 5 × 5, the original image was successively entered into shift registers of 5 lines after a 256-line delay, and the data from each line were successively entered into 5-pixel buffer registers. The mean and variance of the image were calculated independently for the numbers in the window, and the method of calculation was the same as in Part 2. Specifically, to reduce the computational volume and image delay, the standard deviation and mean values of the 16 domain pixels highlighted in red were used to decrease the computational volume and image delay. The mean value of the 16 data points was computed by simply shifting the sum value 4 bits to the right instead of using a divider, and the local values of a and b were calculated by Equation (4). This step used pixel shift registers of 5 lines + 25 pix and one divider; the image’s delay was 3 lines + 36 pix.

(2) Image layering. To create a 5 × 5 real-time image window, a and b were entered into 5-line shift registers, and the data from each line were entered into 5-pixel buffer registers. To calculate a and b and arrive at the mean values of Ma and Mb, the same 16 neighborhood values were chosen. Finally, the background layer image *Base* was calculated by applying Equation (4); the detailed layer image was *Detail* = *q* − *Base*. To match the alignment of a and b, this step specifically called for a delay of 3 lines + 8 pix of the original image. The image delay in this stage was 3 lines + 8 pixel clocks or 3680 clocks, and it used pixel shift registers of 13 line + 58 pix, as well as various other auxiliary computing resources.

All of Part 3 required pixel shift registers of 15 lines + 75 pix, or 0.0147 Mb of Block RAM, a divider, and several auxiliary calculation resources; the image’s delay was 6 lines + 44 pixel clocks or 7388 clocks.

Part 4: The fourth part involved the calculation of the global brightness guide map. It involved the standard computation of the global image as described in Part 2. To reduce the amount of calculation, downsampling and summing were performed in the process of image summation, following the method of discarding one out of every three pixels. Meanwhile, the standard deviation of the previous frame was used as a parameter to calculate the guide image of the current frame in the process of continuous video processing. Finally, Equation (5) was used to calculate the global brightness guide image of the frame in real time. A divider, a root calculator, and some more processing resources were needed for this section, and the image’s delay, which was caused by the divider, was only 26 clocks.

Part 5: The fifth part was to calculate the final output image, which included three main steps:

(1) Determining the brightness value of the original image under different local block stretching factors, while 192 local blocks were calculated simultaneously to yield B1, B2, …, Bn. The calculation used Equation (6), and the delay was only one clock.

(2) Determining the weight value using the formula in Equation (2). The different local block weights were based on the distance of the actual pixel from each local block, and the distance could be obtained by extrapolating the row counter. In this step, exponential calculation was involved, so optimization was carried out as follows

(15) $$\exp(-\frac{d_{\left(i,j)(x,y\right)}{}^{2}}{\delta^{2}})=\exp(-\frac{d_{\left(i)(x\right)}{}^{2}+d_{\left(j)(y\right)}{}^{2}}{\delta^{2}})=\exp(-\frac{d_{\left(i)(x\right)}{}^{2}}{\delta^{2}})\times \exp(-\frac{d_{\left(j)(y\right)}{}^{2}}{\delta^{2}})=ex\times ey$$

where $d_{\left(i,j\right)\left(x,y\right)}\;$denotes the Euclidean distance from a pixel with the coordinates $\left(i,j\right)$ to the center of the local block with the coordinates $\left(x,y\right)$, $d_{\left(i\right)\left(x\right)}$ denotes the horizontal distance from a pixel with the coordinates $\left(i,:\right)$ to the center of the local block with the coordinates $\left(x,:\right)$, and $d_{\left(j\right)\left(y\right)}$ denotes the vertical distance from a pixel with the coordinates $\left(:,j\right)$ to the center of the local block with the coordinates $\left(:,y\right)$. According to $\delta^{2}$, when $d_{\left(i\right)\left(x\right)}=256\;$or $d_{\left(j\right)\left(y\right)}=256$, ex ($ex=\exp(-\frac{d_{\left(i)(x\right)}{}^{2}}{\delta^{2}})$) and ey ($ey=\exp(-\frac{d_{\left(j)(y\right)}{}^{2}}{\delta^{2}})$) almost decay to zero, so they can be ignored. Therefore, ey and ey have at most 256 cases, or 256 values at x,y=0,1,…255. Therefore, to avoid exponential calculations in the FPGA, the process calculated the value at x,y=0,1,…255  and saved it in the Block RAM. During the real-time computation, only the lookup table was needed to obtain ex and ey, and only a simple multiplication operation needed performing to obtain the weights. The process of reading the lookup table only delays this step by one clock but requires a Block RAM with a depth of 256 and a width of 16 bits.

(3) The weighted average calculated the final B. The final B was obtained by weighting B1, B2, …, Bn. This step involved a divider, delayed by 26 pixel clocks.

(4) The last step was to calculate the final image. The detailed layer image was adaptively enhanced and delayed before being added to the background layer image to obtain the final image. The total delay was 55 clocks, with the detailed layer requiring a delay of 53 clocks.

Therefore, Part 5 requires two dividers, 0.0039 Mb of Block RAM, and 55 clocks of image delay.

Overall, the entire process used 2.2554 Mb of Block RAM, five dividers, a root calculator, and some other auxiliary computational resources, with a total image delay of 0.018 s. The specific resource utilization of the FPGA is shown in Table 2.

**Figure 10:**
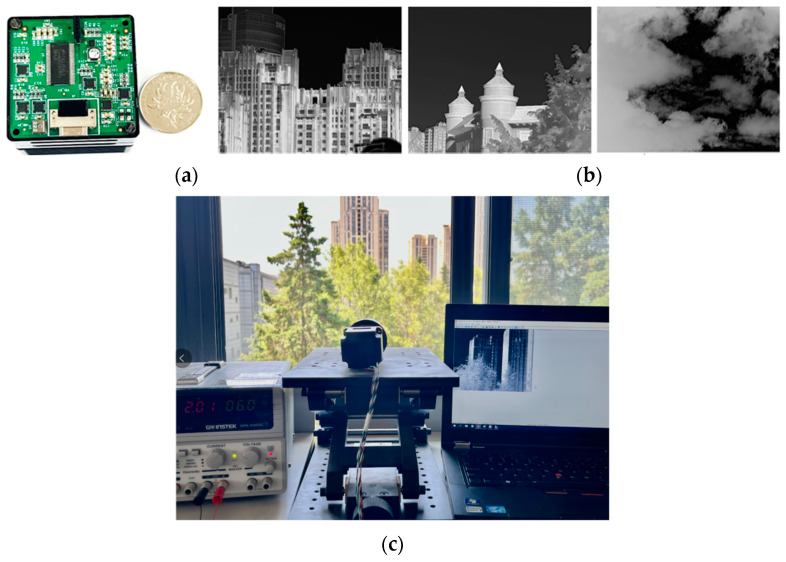
Infrared imager and intended display effects. (**a**) The infrared imager; (**b**) The different display effects in different scenes; (**c**) experimental environment.

The proposed algorithm can be widely used in infrared imaging modules, which can be applied to various military and civilian thermal imagers and scientific research equipment. The infrared imager used in this study was small in size, as shown in Figure 10a, and the effect is shown in Figure 10b. It achieved good imaging results for different scenes. Figure 10c is a schematic diagram of the imaging module mounted on the optomechanical system.

## 4. Conclusions

In this study, an algorithm for enhancing the contrast and compressing the dynamic range of infrared images with local optimal contrast was proposed. The focus was to strengthen and improve the following aspects:

(1) Low-delay image processing technology. Most of the algorithms used for conventional background layer image processing are Gamma correction algorithms or histogram-based equalization algorithms. Among them, Gamma-based correction algorithms are generally effective for comparing images, while histogram-based or improved algorithms must count one frame before outputting the final image, which means that at least one whole image will be delayed and there may be phenomena such as image overenhancement. In particular, in continuous video sequences, we found in experiments that when the histogram statistics of the previous frame are used as the current mapping curve, the brightness of the continuous image will flicker due to the mismatch between the real image and the mapping curve. Therefore, an image processing algorithm with a low delay (less than one frame) focuses on the compression of the background layer, so this study proposed a local contrast enhancement framework based on guided global compression of the image.

(2) Minimal storage resources. One of the most crucial components of FPGA-based image processing algorithms is the Block RAM. It is necessary to keep more than one frame of image data when an image processing algorithm has a delay of more than one frame. The block-based local optimal contrast stretching algorithm proposed in this study uses fewer storage resources and only needs two parameters for each local block.

(3) The design of the algorithm structure of the pipeline and the efficient implementation of the algorithm based on FPGA. The pipeline of the algorithm’s structure is conducive to the implementation of FPGA; however, exponentiation, division, and other calculations require a significant amount of the FPGA’s resources; therefore, this study designed the entire algorithm in accordance with the pipeline and optimized the calculations in the algorithm to facilitate the efficient operation of the FPGA.

(4) Consideration of both global and local contrast. Previous algorithms based on local histograms are prone to block effects, and global class-based algorithms have poor local contrast. Based on this, this study improved the local contrast while ensuring that the global contrast closely resembled the original image. Specifically, the global background layer’s compressed image is used as the guide image to achieve the optimal enhancement of local contrast.

In general, in order to effectively suppress local noise and the halo effect while maintaining both global and local contrast, the proposed algorithm combines local information with global information and uses the global contrast compression map as the guide image. Meanwhile, this study used infrared image with a resolution of 1024 × 768 as an example for optimization and implementation of the FPGA-based algorithm, and the entire algorithm used 2.2554 Mb of Block RAM, five dividers, one root calculator, and some other auxiliary computing resources, with a total image delay of 0.018 s. Finally, the image processing results of different scenes showed that the algorithm proposed in this study had good results in rich scenes, scenes with small targets, and indoor scenes. At the same time, the algorithm has low complexity and low delay, which is beneficial for its application to scenes with high requirements in real time.

## Figures and Tables

**Table 1:** Evaluation results of the image quality of different algorithms.

	Index	AGC	HE	CLAHE	GF-DDE	BF-DRP	Proposed Method
**Scene 1**	**RMS**	31	49	42	**51**	50	48
	**Entropy**	6.989	**7.991**	7.799	7.711	7.929	7.744
	**SSIM**	1	**0.8061**	0.561	0.6092	0.6980	0.6168
	**Tenengrad**	3.257	7.581	19.642	14.813	11.8181	**28.561**
	**Time (s)**	**0.0020**	0.6856	4.6780	0.9384	24.8654	0.3168
**Scene 2**	**RMS**	1	31	16	22	32	**33**
	**Entropy**	2.972	**7.990**	7.322	7.609	7.888	7.393
	**SSIM**	1	**0.4653**	0.344	0.408	0.386	0.454
	**Tenengrad**	0.283	15.371	29.906	23.515	18.754	43.940
	**Time (s)**	**0.0024**	0.6670	4.6160	0.9418	25.3805	0.3651
**Scene 3**	**RMS**	3	31	8	28	**32**	17
	**Entropy**	4.587	**7.953**	6.278	7.142	7.924	7.378
	**SSIM**	1	0.556	**0.774**	0.483	0.656	0.547
	**Tenengrad**	1.411	7.625	6.042	7.878	5.489	**9.661**
	**Time (s)**	**0.0018**	0.6843	4.6874	0.9400	23.3256	0.3358

The results of running the software on a computer and its implementation on the FPGA were consistent.

**Table 2:** Utilization report.

Slice LUTs (total: 134,600)	39,045 (29%)
Slice registers (total: 269,200)	38,919 (14.46%)
Block RAM (total: 365)	105 (28.77%)
DSPs (total: 740)	319 (43.1%)

## Data Availability

Not applicable.

## Abbreviations

**Abbreviations**	**Full Name**
ADC	Analog to digital converter
IR	Infrared radiation
HDR	High dynamic range
PHE	Plateau histogram equalization
CLAHE	Contrast-limited adaptive histogram equalization
GDHDRC	Gradient domain-based high dynamic range image compression
FPGA	Field-programmable gate array
CPU	Central processing unit
DRCE	Dynamic range compression and enhancement algorithm
LOC	Local optimal contrast
BF-DRP	Bilateral filter and dynamic range partitioning
LPF	Low-pass filter
GF	Guided filter
LEPF	Local edge-preserving filter
DDE	Digital detail enhancement
pix	Pixel
AGC	Adaptive gain control
